# Induction of Radiata Pine Somatic Embryogenesis at High Temperatures Provokes a Long-Term Decrease in DNA Methylation/Hydroxymethylation and Differential Expression of Stress-Related Genes

**DOI:** 10.3390/plants9121762

**Published:** 2020-12-13

**Authors:** Ander Castander-Olarieta, Cátia Pereira, Ester Sales, Mónica Meijón, Isabel Arrillaga, María Jesús Cañal, Tomás Goicoa, María Dolores Ugarte, Paloma Moncaleán, Itziar A. Montalbán

**Affiliations:** 1 Department of Forestry Science, NEIKER, 01192 Arkaute, Spain; acastander@neiker.eus (A.C.-O.); catia.pereira@student.uc.pt (C.P.); 2 Center for Functional Ecology, Department of Life Sciences, University of Coimbra, 3000-456 Coimbra, Portugal; 3 Departament of Ciencias Agrarias y del Medio Natural, Instituto Universitario de Ciencias Ambientales, Universidad de Zaragoza, Escuela Politécnica Superior, 22071 Huesca, Spain; esales@unizar.es; 4 Plant Physiology, Department of Organisms and Systems Biology and University Institute of Biotechnology (IUBA), University of Oviedo, 33006 Oviedo, Spain; meijonmonica@uniovi.es (M.M.); mjcanal@uniovi.es (M.J.C.); 5 Departamento de Biología Vegetal, Facultad de Farmacia, Instituto BiotecMed, Universidad de Valencia, 46100 Burjassot, Spain; isabel.arrillaga@uv.es; 6 Department of Statistics, Computer Science and Mathematics, Universidad Pública de Navarra, 31006 Pamplona, Spain; tomas.goicoa@unavarra.es (T.G.); lola@unavarra.es (M.D.U.); 7 INAMAT2 (Institute for Advanced Materials and Mathematics), Universidad Pública de Navarra, 31006 Pamplona, Spain

**Keywords:** epigenetics, 5-hydroxymethylcytosine, 5-methylcytosine, heat, heat shock protein, memory, *Pinus radiata*, priming, somatic embryo, somatic plant

## Abstract

Based on the hypothesis that embryo development is a crucial stage for the formation of stable epigenetic marks that could modulate the behaviour of the resulting plants, in this study, radiata pine somatic embryogenesis was induced at high temperatures (23 °C, eight weeks, control; 40 °C, 4 h; 60 °C, 5 min) and the global methylation and hydroxymethylation levels of emerging embryonal masses and somatic plants were analysed using LC-ESI-MS/ MS-MRM. In this context, the expression pattern of six genes previously described as stress-mediators was studied throughout the embryogenic process until plant level to assess whether the observed epigenetic changes could have provoked a sustained alteration of the transcriptome. Results indicated that the highest temperatures led to hypomethylation of both embryonal masses and somatic plants. Moreover, we detected for the first time in a pine species the presence of 5-hydroxymethylcytosine, and revealed its tissue specificity and potential involvement in heat-stress responses. Additionally, a heat shock protein-coding gene showed a down-regulation tendency along the process, with a special emphasis given to embryonal masses at first subculture and ex vitro somatic plants. Likewise, the transcripts of several proteins related with translation, oxidative stress response, and drought resilience were differentially expressed.

## 1. Introduction

Epigenetic mechanisms refer to all the mitotically and/or meiotically heritable changes in patterns of gene expression that occur without alterations in DNA sequence [1]. The regulation of gene expression is tightly influenced by the accessibility of genes for the transcriptional machinery, and the packaging grade of chromatin is fundamental during these processes [2]. Multiple mechanisms have been found to define the distinct chromatin states and accessibility of regulatory elements. Among them, covalent modifications (acetylation, methylation, phosphorylation, ubiquitination and sumoylation) of histone proteins tails, addition of histone variants, the position and spacing of nucleosomes, and cytosine methylation of the DNA are the main targets of current epigenetic studies [3]. Moreover, non-coding RNAs, including small RNAs, are currently gaining special interest as novel epigenetic regulators influencing the distribution of epigenetic marks and the expression of specific genes at both transcriptional ad post-transcriptional levels [4].

DNA methylation is the most studied epigenetic mark due to its occurrence in plants and mammals, its stability, and its role in gene regulation and genome structure maintenance through transposon silencing [5]. Methylation is site specific and usually occurs in the fifth carbon position of cytosines in the following sequences: CG, CHG, and CHH (where H = A, T or C) [6]. Although DNA methylation has frequently been linked with gene expression repression, its specific location in, for example, CG gene body sequences, has most often been associated with transcriptional activity [7].

The recent progress in genome-wide analysis of epigenetic marks has demonstrated that these mechanisms are essential for the adaptation of plants to changing environments and stress conditions through phenotypic plasticity [8]. This adaptive response is based on the ability to produce different phenotypes in response to different environments, and results of especial relevance in the case of non-mobile and long-living organisms like trees under the current climate change situation [9]. In this sense, variations of DNA methylation levels have been documented under different stress conditions including heat [10,11].

Most of the epigenetic marks activated under stress events are reverted when the environmental constrain is no longer present. However, plants can maintain part of those marks developing the capacity to remember the stress episode (stress priming) and establishing an epigenetic memory that leads to both temporary or sustained structural, genetic, and biochemical modifications that prepare plants to respond more efficiently to future stress exposures [12]. Furthermore, in some cases, a crosstalk among different stimuli can occur, leading to multiple stress memory acquisition (cross-priming) [13].

There are multiple examples in nature that support this phenomenon. Vernalization, in which cold exposure of winter annual plants synchronizes flowering to the optimal season, is a clear example [14]. Memory of pathogen attack, formerly referred to as systemic acquired resistance, is well documented [15,16,17], and the chemical priming of seeds to enhance stress tolerance and pathogen resistance of young plants after germination is a usual agronomic practice [3]. Some of the best-known examples of epigenetic memory in forest tree species are found in Norway spruce, for which the environmental conditions during both zygotic and somatic embryogenesis (SE) seem to modulate the timing of bud set and bud burst of plants years later [18]. In the case of poplar trees, differences in DNA methylation of stress-responsive genes and hormonal pathways have been observed in winter-dormant shoot apical meristems after drought events during the vegetative period [19].

In addition to the common methylation of cytosine, in recent years other modifications of cytosine at the same position, such as the oxidized form 5-hydroxymethylcytosine (5hmC), have gained special attention because of their role as demethylation intermediates and transcriptional regulators during many cellular and developmental processes in mammals and fungi [20]. Although the presence of 5hmC in plants is still ambiguous, increasing evidence supports its non-casual enzymatic origin [21], and its specific and conserved location in several plant genomes, potentially contributing to plant development and homeostasis control [22]. Moreover, the latest studies have revealed non-negligible quantities of this molecule in the DNA of a conifer species [23].

In previous studies we have observed that the application of high temperatures during initiation of *Pinus radiata* and *Pinus halepensis* SE modulates not only the success of the different stages of the process, but also the morphology and biochemical status (hormones, metabolites) of embryonal masses (EMs) and somatic embryos (Se’s) [24,25,26,27]. Moreover, those changes can have long-lasting effects determining the ex-vitro behaviour of somatic plants years later at both control and stress conditions [25].

As a result, in this study, we have tried to evaluate if all those changes can be attributed to the establishment of an epigenetic memory based on specific modifications of cytosine residues by heat. To this aim, high liquid chromatography (HPLC) coupled to mass spectrometry has been employed, which allows a sensible and precise identification of the different nucleotides, including 5mhC, and requires little amount of genomic DNA starting material [28]. In parallel, the differential expression of putative stress-related genes throughout the SE process has been analysed to assess if the observed epigenetic changes have provoked modifications at the transcriptome level.

## 2. Results

### 2.1. Global DNA Methylation/Hydroxymethylation Analysis

Global DNA methylation analysis in T2 samples (proliferating EMs) revealed high levels of 5-methylcytosine (5mC) in all treatments (around 40%), but no significant differences among them. However, the differences observed were on the verge of statistical significance (*p* = 0.0934), being the treatment at 60 °C for 5 min the one presenting the lowest global methylation values. These levels in the other two treatments, including the control temperature of 23 °C, were slightly higher, being 40 °C for 4 h treatment, the one presenting the highest difference (3.03%) with respect to the treatment with the lowest values (60 °C for 5 min) (Table 1).

Although 5hmC was undetectable in most of the T2 samples, a fact that made impossible the comparison among treatments, in the sample presenting the highest total cytosine pool very low levels of 5hmC could be detected (0.004%; data not shown). As a result, we can assume that this molecule was present in the rest of the samples, but its levels were under the limit of detection due to a lower content of total cytosine residues.

Regarding the results from T5 samples, the observed global DNA methylation (GDM) rates were similar to those from EMs (around 40%), but in this case statistically significant differences were observed among treatments. The pattern was similar to the one observed previously; the levels of 5mC were considerably lower at treatment 60 °C for 5 min (almost 3% if compared with the other two treatments), but in this case both control treatment at 23 °C and treatment at 40 °C for 4 h showed very similar GDM rates (Table 1).

In opposition to the results obtained from proliferating EMs, in one-year-old somatic plant needles the levels of 5hmC were detectable in almost all the samples analysed (83.3%). Despite being considerable low rates, the values observed were more than twice as high as those from EMs. Considering the effects of temperature, significant differences were observed among treatments. Hydroxymethylation rates were appreciably lower in both groups of samples originating from EMs subjected to heat-stress. In this case, the levels of both treatments (40 °C, 4 h, and 60 °C, 5 min) showed very similar values. The same pattern was detected when analysing the percentage of hydroxylated cytosine forms with respect to the total amount of modified cytosine bases (5hmC/5mC × 100). On this subject, both treatments at high temperatures were the ones with the lowest 5hmC rates, and the values between them were also very similar (Table 1).

### 2.2. Expression Pattern of Stress-Related Genes

The relative expression analysis of genes involved in heat and drought stress responses revealed that the application of high temperatures during induction of SE in radiata pine resulted in the differential expression of certain genes with respect to the control treatment of 23 °C throughout the entire process and in somatic plants more than one year later. The results also showed that in most of the cases the differential expression of the genes changed along the different stages of SE, not following a defined pattern.

**Figure 1:**
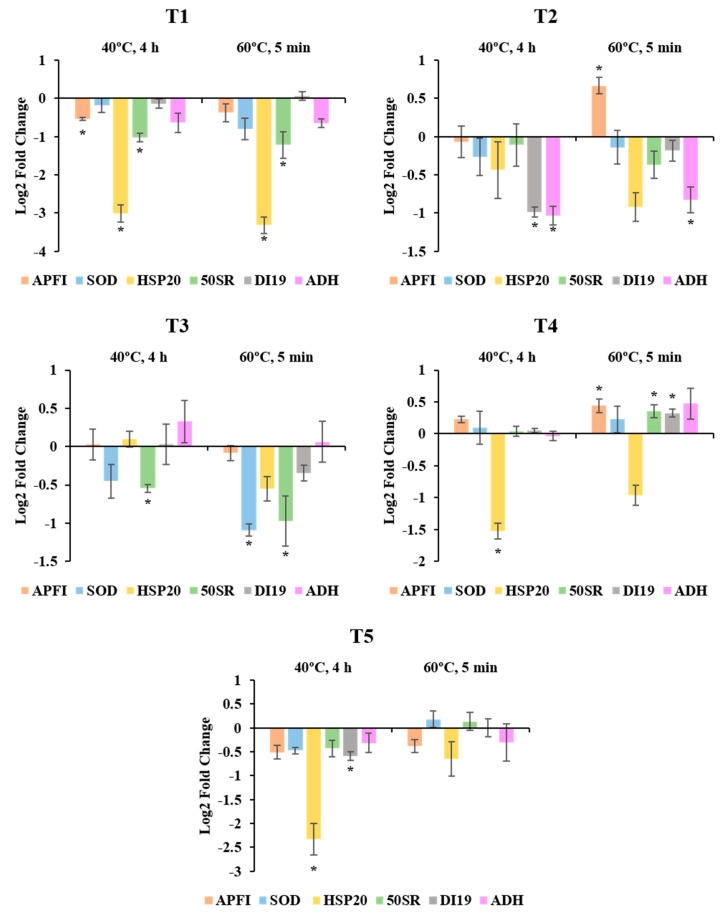
Fold-relative gene expression of six stress-related genes (*APFI*, *SOD*, *HSP20*, *50SR*, *DI19* and *ADH*) along the different stages of *Pinus radiata* somatic embryogenesis (T1, T2, T3, T4 and T5) between samples originating from different temperature conditions during induction (23 °C, 8 weeks; 40 °C, 4 h; 60 °C, 5 min). Data are presented as mean values ± SE. * Denotes significant differences at *p* < 0.05 among high temperature treatments with respect to the control temperature of 23 °C.

For samples at T1, significant differences were found for three of the six genes analysed: *APFI*, *HSP20* and *50SR* (Figure 1; T1). The most evident changes were observed for *HSP20*. In both 40 °C and 60 °C treatments, the relative expression of this gene was considerably lower than in control temperature of 23 °C, presenting high fold change values. The same pattern was observed for *50SR* (lower expression at both 40 °C and 60 °C treatments), but with lower fold changes. In the case of the transcription factor *APFI* however, the differences were only significant for the 40 °C treatment, and its expression was just slightly lower than that of the control treatment.

Samples at T2 showed differential expression of *APFI*, *DI19,* and *ADH* (Figure 1; T2). As in T1, the gene *APFI* presented small significant differences, but at this stage its expression increased in EMs originating from the treatment at 60 °C for 5 min, while no differences were observed in those from the 40 °C treatment. *ADH* and *DI19* showed a decreasing pattern, with significant differences at both high temperature treatments for *ADH* and only at 40 °C for *DI19*. It is also noticeable that despite not being significant, the expression of *HSP20* at this stage was also lower at high temperatures, especially at 60 °C for 5 min. In any case, fold changes were lower if compared with T1.

In mature Se’s (T3), genes *50SR* and *SOD* presented lower expression values at high temperatures than at control conditions (Figure 1; T3). Regarding *50SR*, the expression pattern was similar to the one observed at T1, with significant differences in both treatments, while in the case of *SOD* significant differences were only detected at 60 °C. As in T2, fold changes were low for all the analysed genes.

At T4, *HSP20* presented the most noticeable results. Although both treatments at high temperatures showed a decreasing tendency, only the expression levels at 40 °C for 4 h were statistically significant. Certain genes were up-regulated in 60 °C treatment (*APFI*, *50SR* and *DI19*), but their fold changes were very low (Log2 Fold change < 0.5). With the exception of *HSP20*, the relative expression of the rest of the genes was very similar between control conditions and high temperatures (Figure 1; T4).

Very similar results were obtained at T5, but with an enhanced response of *HSP20*. As in in vitro plantlets, the expression of this gene was notably down-regulated in needles of one-year-old somatic plantlets at 40 °C. In this case, the fold change was higher, reaching values similar to those from T1. At 60 °C treatment however, the differences observed for *HSP20* were low and not significant. Following with this treatment, it is worth mentioning that the increase observed for *APFI*, *50SR* and *DI19* at T4 were no longer present at this stage, while at 40 °C treatment *DI19* showed slightly lower significant values (Figure 1; T5).

## 3. Discussion

Epigenetics has been demonstrated to be essential for long-lifespan organisms with complex life-cycles like forest tree species, regulating many developmental and stress-response processes. During the annual cycle, for example, different developmental transitions like bud set in autumn and bud burst in spring are governed by changes in DNA methylation patterns [29]. Moreover, these epigenetic alterations and the establishment of epigenetic marks strongly contribute to the development of phenotypic plasticity and environmental stress memory through the differential expression of specific genes and the regulation of transposable elements mobility [5]. Despite this fact, only limited studies have been implemented with trees and we still lack information about the multiple regulatory layers connecting epigenetic variations, gene expression, and phenotypic traits in trees.

The combination of natural or artificially-induced epigenetic diversity exploitation and selection of epigenetic marks could provide fitness advantage to clonal trees with limited genetic diversity under the on-going climate change situation, highlighting the potential role of epigenetics in tree improvement and breeding [12]. In this regard, embryo development and seed maturation seem to be crucial periods for the formation of stable epigenetic modifications [30]. This could be used as a valuable tool during clonal propagation systems (i.e., SE) to produce epigenetic variants by the application of controlled stresses early in the life of the plant [31].

Following this idea, the GDM analysis carried out in this study during radiata pine SE revealed that proliferating EMs were highly methylated (>37% in all treatments) if compared with several studies carried out in EMs of *P. radiata* and other *Pinus* species. In [2] they observed GDM rates lower than 5% in similar-aged EMs of radiata pine and results in *Pinus nigra* underlined that the formation of bipolar structures in EMs can only be obtained when 5mC levels are not higher than 18% [32]. Nonetheless, micromorphological analyses carried out in our laboratory confirmed the presence of polarised structures presenting different developmental and organizational stages under similar culture conditions in radiata pine [24].

Induction of SE and cell dedifferentiation is usually accompanied by a drastic hypomethylation of DNA, enabling the expression of key genes involved in SE such as *WUS* and *BBM* [33]. Moreover, embryogenic potential is usually correlated with low 5mC levels, as observed in many studies with both angiosperms and gymnosperms where embryogenic cell lines (ECLs) able to develop the whole embryogenic program and produce plants showed lower DNA methylation rates than non-embryogenic calli [34,35].

However, some studies suggest that the GDM rate of EMs could be species and developmental stage specific. In two *Acca sellowiana* accessions, [36] reported levels of DNA methylation ranging from 22.6% to 44.3% during 30 days of culture. [37] observed methylation rates of 45.8% in EMs of a hybrid larch (*Larix × eurolepsis*) and showed that when those EMs were subjected to maturation, methylation drastically increased up to 61.5% in the first week, followed by a decrease to 53.4% in mature cotyledolary Se’s. Likewise, during microspore SE in *Brassica napus* a decrease in DNA methylation occurs, while during embryo differentiation GDM increases [38]. Several authors even highlighted the association between increased methylation levels during Se’s differentiation, high cell division rates, and activation of embryogenic gene expression program [39]. As a result, the methylations rates observed in radiata pine EMs in this study could be associated with the specific developmental stage at which they were collected.

The results obtained in needles of one-year-old somatic plants are in agreement with previous studies carried out in radiata pine trees [40,41]. These authors observed specific methylation rates associated with aging and phase-change in this species, and reported GDM of around 30% in basal portions of juvenile radiata pine tree needles, while those rates increased up to almost 60% in the apical areas. In the current experiment full needles were analysed, so the methylation rates detected correlate with those studies. Interestingly, GDM levels in needles were similar to those observed in EMs. It would be interesting to study the evolution of GDM after the first subculture of EMs, to confirm whether methylation increases progressively with each subculture period, and also to observe what happens during Se’s formation and plant conversion.

Regarding the effect of temperature on GDM levels during induction of SE, significant differences were detected among treatments at plant level. Although not significant, in EMs, the 60 °C for 5 min treatment provoked a decrease in the content of 5mC, while moderate temperatures and long-exposures slightly increased those levels. In ex vitro plants, those effects were more evident. The pattern observed in EMs was attenuated at the highest temperature (60 °C), presenting a significant GDM decrease of around 3% when compared to the other treatments. In *Arabidopsis*, [42] reported that a chemically-induced 3% decrease in DNA methylation led to the differential expression of 1794 transcripts.

Although many studies have underlined the responsiveness of the methylome to heat, changes of DNA methylation under this stress seem not to follow a consistent trend if observed the published data, since different species and cell types appear to respond in different ways. Exposure of *Arabidopsis* plants to heat resulted in increased GDM [43]. The same trend was observed in Norway spruce seedling originating from a warm embryonic environment [44,45] also observed differences in the content of 5mC of Scots pine embryos from contrasting habitats. On the other hand, in cotton anthers and oilseed rape cultured microspores, heat led to hypomethylation of the DNA [11,46].

Although preliminary, these results could be connected with the developmental and drought resilience changes observed among somatic plants originating from different induction temperature treatments in a previous study [25]. There, we observed that differences temperature pulses at this stage provoked variations in the growth rates of plants years later in the greenhouse. In this regard, [47] showed that the application of the demethylating agent 5-Azacytidine led to a reduction in the growth rate of oak seedlings. In addition, some other studies are highlighting the interconnection of the methylation status of plants and their capacity to cope with stress, i.e., drought [48]. As a result, further analyses addressing the specific distribution of methylated regions are required to confirm our hypothesis and shed light on the molecular mechanisms leading to those phenotypic variations.

The use of HPLC coupled to mass spectrometry in this study enabled the identification of 5hmC in both EMs and somatic plant needles of radiata pine, although at very low concentrations. It is noticeable however, that radiata pine genome is 23 billion base pairs, eight times the size of the human genome. Consequently, even if the percentage of 5hmC is not so high, the amount of hydroxylated cytosines could be relevant in terms of total number. As far as we know, this is the first report confirming the presence of this oxidised form of 5mC in pines, and the second in conifers [23]. In fact, [45] reported no traces of this molecule in zygotic embryos and megagametophytes of Scots pine using a CG-MS method.

Despite being considered the sixth nucleobase in DNA and its demonstrated functionality in mammals [20], researchers are still doubtful about its existence and biological function in plants. As a matter of fact, 5hmC has been found at very low quantities in plant genomes. This, together with the existence of DNA glycosylases in plants that can cleavage 5mC directly from the genome [49] and the lack of enzymatic homologues in plants for the mammal readers and writers of 5hmC (TET/UHRF2) [50], suggest that 5hmC may be the end product of DNA damage, being involved in passive demethylations processes [20]. However, some studies defend its enzymatic origin [21,23] and due to its high abundance in transposable elements located in heterochromatin regions [22] and during specific developmental stages [21], it may be involved in heterochromatin formation and maintenance of genome stability together with 5mC [51].

Supporting this idea, we found different concentrations of 5hmC in EMs and needles of one-year-old somatic plants. The hydroxymethylation levels in EMs were considerably lower than in needles, being almost undetectable in most of the samples. However, methylation levels were similar in both tissue types. Accordingly, in human cells, [52] found that the content of 5hmC is highly variable and does not necessarily correlate with the content of 5mC. In line with these findings, we also observed decreased levels of 5hmC in needles of somatic plant originating from high temperatures. Although methylation rates presented the same tendency at 60 °C treatment, at 40 °C changes in methylation did not correlate with those of 5hmC. There are not studies addressing the role of 5hmC under stress conditions in plants, but based on these results we can hypothesize that this modification of cytosine may take part in numerous cell processes, including stress responses, not only as a passive intermediate of demethylation but also as a potential epigenetic mark in plants.

Together with changes in methylation and hydroxymethylation rates, the present study confirmed the differential expression of several stress-related genes along the phases of SE in radiata pine. Even though a clear pattern was not maintained throughout the process, some points were worth noting. As a general trend, the gene coding for a heat shock protein (*HSP20*) was down-regulated at high temperatures in most of the developmental stages analysed, presenting the highest fold changes at early stages in EMs and at plant level. Heat shock proteins (HSP) are essential during exposure to heat stress by preventing proteins from denaturation, and their sustained accumulation enhances thermo-tolerance [53]. Responsiveness under other stress conditions like drought has also been documented [54].

The long term down-regulation of HSP at high temperatures in this study may correlate with the decreased drought-resilience observed in previous studies in plants originating from high temperatures [25]. In fact, although the expression of genes encoding HSP tend to increase under heat conditions, several authors have reported that selective autophagy mediates the specific degradation of HSP at later stages of thermo-recovery phase, compromising the long-term acquired HSP memory [55,56].

Alternatively, other authors have postulated that all types of HSP may not be involved in regulation of heat acclimation and have reported unique transcription patterns for several HSP homologs [57]. Furthermore, many studies have reported poor transcript/proteins correlations. For instance, in heat-primed azalea plants recover to control temperature, transcripts of HSP decreased rapidly in a short time, while proteins maintained a sustained accumulation for a long period, therefore conferring resistance to subsequent stress events [58]. As a result, it would be of special interest to combine the results from this experiment with proteomic analyses to confirm our hypothesis.

In proliferating EMs and Se’s, transcripts encoding enzymes involved in oxidative stress (*ADH* and *SOD*) were found at lower concentrations. Although these enzymes are required to detoxify compounds resulting from heat-stress at short-term exposures, their accumulation during long-term acquired tolerance or acclimation seem not to be significant [59]. The same pattern was observed for the ribosomic protein *50SR* in EMs at initiation and in Se’s. During Se’s formation, protein homeostasis and synthesis of reserve proteins are of special relevance [60]. Consequently, alterations in the levels of proteins involved in translation like *50SR* could have important effects for the success of the process, even determining the morphology of Se’s, as observed in previous studies [24]. Moreover, the biological function of this *50SR* has also been linked to cytokinins [61], and its down-regulation as a consequence of high temperatures could be an explanation for the decrease of most of the cytokinin types analysed in [25,26] under the same temperature conditions.

In spite of being previously categorised as an excellent candidate biomarker under heat-stress in radiata pine, and the demonstrated relevance of transcriptional control during thermotolerance [59], the transcription factor *APFI* did not show a clear pattern in our study and its gene expression differences presented very low fold changes. It is noticeable that [59] only observed its protein accumulation at early stress-response stages. Similar results were obtained for *DI19*. DI19 family of proteins are a novel type of Cys2/His2 zinc-finger proteins and their overexpression results in drought-sensitive phenotypes in *Arabidopsis* [62]. However, some studies suggest that post-translational modifications of these proteins may be important for regulating their function [63], and thus, further research is required to assess whether the observed transcript profile changes are reflected in the phenotype.

In conclusion, this study confirmed that high temperatures at early stages of radiata pine SE can provoke long-term changes in the methylation status of the resulting somatic plants, revealing the formation of a stable epigenetic memory. We also detected for the first time in a pine species the presence of 5hmC, and observed that its concentration can fluctuate according to the tissue type or to environmental factors like temperature. All these alterations could be related with the sustained differential expression of several stress-related genes along the whole embryogenic process, with a special emphasis on the role of HSP.

## 4. Materials and Methods

### 4.1. Plant Material and Heat Stress Experiment

Seeds of *P. radiata* were extracted from green female cones collected in June 2018 from five genetically different open-pollinated trees, and surface sterilized following [64]. Megagametophytes enclosing immature zygotic embryos were excised out aseptically and placed horizontally onto Petri dishes containing Embryo Development Medium (EDM) [65], supplemented with 3.5 g L^−1^ gellan gum (Gelrite^®^, Duchefa, Haarlem, The Netherlands). At this point, explants were subjected to different temperature and incubation time periods: 23 °C, 8 weeks; 40 °C, 4 h; 60 °C, 5 min. The culture medium was pre-warmed for 30 min before the start of the experiment and at the end all the megagametophytes were kept at 23 °C in darkness. Eight megagametophytes were used per Petri dish and ten Petri dishes per treatment, comprising a total of 1200 megagametophytes, including all mother trees.

After 8 weeks on the same medium, emerging EMs bigger than 3–5 mm in diameter were separated from the megagametophytes and subcultured to fresh EDM medium. After 14 days, a small part from each EMs was frozen in liquid nitrogen and stored at −80 °C for subsequent molecular analyses (T1). The other part was transferred to medium of the same composition but 4.5 g L^−1^ gellan gum fortnightly until maturation 4 weeks later. At this point, actively proliferating EMs were selected, and half of the embryogenic tissue was frozen in liquid nitrogen as explained before (T2). The other part was subjected to maturation as described in [66]. Six replicates (Petri dishes) per ECL and 10 ECLs per treatment were used. After 12 weeks, 100 mg of mature and well-formed Se’s were frozen in liquid nitrogen (T3).

The remaining Se’s were placed with root caps pointing downwards on Petri dishes with half-strength macronutrients LP medium (1/2 LP) [67] modified by [68] and supplemented with 2 g L^−1^ activated charcoal and 9 g L^−1^ gellan gum (Difco^®^ Agar granulated, Becton Dickinson, Franklin Lakes, New Jersey, USA) at an angle of approximately 60°. For the first 7 days Petri dishes were placed under dim light (10 µmol m^−2^ s^−1^) and then maintained for 5 weeks under 16-h photoperiod at 100 µmol m^−2^ s^−1^ provided by cool white fluorescent tubes (TFL 58 W/33; Philips, Amsterdam, The Netherlands). Successfully germinated seedlings were subcultured to glass jars (diameter 59 and height 66 mm, Sigma-Aldrich, St. Louis, Missouri, USA) with medium of the same composition.

After 6 weeks, part of the seedlings that had developed a proper root system and new non-cotyledonary needles were entirely frozen in liquid nitrogen (T4) and the rest transferred *ex vitro* to 43 cm^3^ pots containing blond peat moss (Pindstrup, Ryomgård, Denmark): vermiculite (8:2, *v*/*v*) in a greenhouse under controlled conditions as described in [25]. Growing saplings with more than 5 cm height were transplanted to bigger pots with new substrate of the same composition but adding 3 g L^−1^ Osmocote^®^ Topdress fertilizer (Everris, Geldermalsen, The Netherlands) and watered regularly for one year. Then, needles form the apical area of the somatic plants were frozen in liquid nitrogen (T5) and stored at −80 °C until DNA and RNA extractions. At this point plants presented similar phenotypic traits (colour and length of needles, number of shoots, etc.) and no differences in the mean height was observed among plants originating from different temperature treatments (23 °C: 32.3 ± 3 cm; 40 °C: 32.2 ± 2.5 cm; 60 °C: 31.5 ± 1.3 cm).

### 4.2. Global DNA Methylation/Hydroxymethylation Analysis

Genomic DNA was extracted from samples collected at T2 and T5 (proliferating EMs and needles of one-year-old somatic plants growing in the greenhouse, respectively). For T2 samples five ECLs were used per treatment and 15 mg of lyophilized tissue was used as starting material, while for T5 samples three ECLs per treatment and two clones per ECL were used (a total of six samples per treatment). As starting material 100 mg of fresh tissue grounded in liquid nitrogen to a fine powder was employed. All samples were extracted in 800 µL preheated (60 °C) buffer containing 2% CTAB, 1.4 M NaCl, 20 mM EDTA, 100 mM Tris-HCl, 2% PVP (*w*/*v*), 8 mM ascorbic acid and 5 mM DIECA, supplemented with 89 mM β-mercaptoethanol. Samples were incubated at 65 °C for 30 min (gently shaking each 10 min), followed by the addition of 500 µL chloroform/isomylalcohol (24:1, *v*/*v*). After vortexing and centrifugation at 10,000 rpm for 5 min, the aqueous phase was transferred to a new tube and RNA was digested by the addition of 10 μL of RNase A (50 mg/mL, Sigma-Aldrich) at 37 °C for 1 h. Phase separation by chloroform/isoamylalcohol was repeated once and then DNA was precipitated by the addition of 600 µL cold isopropanol (−20 °C) and centrifugation at 11,000 rpm for 10 min at 4 °C. DNA pellet was washed with 1 mL ethanol 50%, centrifuged at 14,000 rpm for 5 min, and the supernatant discarded. Pellets were air-dried and DNA was resuspended in 50 µL ultra-pure water. Quantification was carried out using a Nanodrop^TM^ 2000 (Thermo Fisher Scientific, Waltham, MA, USA).

Genomic DNA was hydrolysed as follows: ten microlitres of DNA containing approximately 1 µg DNA were denaturalised at 100 °C for 2 min and digested by the addition of 1.13 μL sodium acetate 50 mM and zinc chloride 40 mM solution and 2.5 μL nuclease P1 (2.5 U, Sigma-Aldrich). Samples were incubated at 37 °C overnight. Then 2.5 μL Tris buffer (0.5 M, pH 8.3) and 1 μL alkaline phosphatase (0.3 U, Sigma) were added and incubated at 37 °C for 2 h and 30 min. After the addition of 40 μL ultra-pure water and precipitation with 200 μL pure ethanol (−20 °C) plus centrifugation at 15,000 for 15 min (4 °C), supernatants were transferred to low-binding tubes and evaporated using a vacuum-concentrator. Finally, digested free nucleotides were resuspended in 100 μL ultra-pure water.

Methylation and hidroxymethylation levels were analysed on a 1200 Series HPLC system coupled to a 6410 Triple Quad mass spectrometer from Agilent Technologies (Santa Clara, CA, USA). The chromatographic separation was performed on a Zorbax SB-C18 column (2.1 × 100 mm, 3.5 µm, Agilent Technologies). The mobile phase was 11% methanol and 0.1% formic acid in water and 5 μL of samples were injected in the column at a flow rate of 0.1 mL min^−1^. The electrospray ionization source (ESI) was operated in the positive ion multiple reaction monitoring mode (MRM) set to an ion spray voltage of 3500 V, 40 psi for nebulizer and source temperature at 350 °C. The intensity of specific MH^+^→fragment ion transitions were recorded (5mC m/z 242→126, 5hmC m/z 258→142 and C m/z 228→112). Identification of cytosine, 5mC and 5hmC were assessed by injection of commercial standards (5-Methylcytosine & 5-Hydroxymethylcytosine DNA Standard Set, Zymo Research, Irvine, CA, USA) under the same LC-ESI-MS/ MS-MRM conditions. The measured percentage of 5mC and 5hmC in each experimental sample was calculated from the MRM peak area divided by the combined peak areas for 5mC plus 5hmC plus cytosine (total cytosine pool). In the case of 5hmC, its percentage with respect to the total modified cytosine pool was also calculated.

### 4.3. RNA Extraction

Extraction of total RNA from samples at T1 (EMs after first subculture) and T4 (in vitro seedling) was performed using the RNeasy Plant Mini Quit (Qiagen, Venlo, The Netherlands) with slight modifications depending on the type of sample. For T1 samples, three pools of 300 mg from different ECLs were employed for each temperature treatment. In this case samples were grinded directly in the lysis buffer with a plastic piston. For T4 samples, 3 ECLs per treatment and 100 mg of fresh material per sample were employed. To eliminate any residual genomic DNA, RNA samples were treated with Recombinant DNase I (RNase-free, Takara Bio, Shiga, Japan) according to the manufacturer’s protocol. The quantity of isolated RNA both before and after DNase treatment was measured using a Nanodrop^TM^ 2000. RNA integrity and potential DNA contamination were assessed by agar gel electrophoresis.

RNA extractions from T2 (proliferating EMs) and T3 (Se’s) samples were performed using five ECLs per treatment and following the combined protocol described by [69] with modifications. This protocol was used because it enables the simultaneous extraction of proteins, metabolites, and RNA. The results from proteins and metabolites extraction will be discussed in further experiments. 0.8 mL and 2 mL of cold extraction buffer were added to 100 mg of grinded T3 samples and 500 mg of grinded T2 samples, respectively. Then, samples were centrifuged, the supernatant discarded, and pellets were washed with 1 mL and 2 mL of 0.75% (*v*/*v*) β-mercaptoethanol in 100% methanol (for T2 and T3 samples, respectively). After another centrifugation step, the supernatant was discarded, and the washing step was repeated once. Pellets were then air dried and dissolved in 1 mL of pellet solubilization buffer for incubation at 37 °C in a thermal shaker for 20 min. Then, samples were centrifuged, and the supernatants were transferred to silica columns to bind DNA. After 1 min of incubation, columns were centrifuged, and the flowthrough was mixed with 300 μL and 600 μL of acetonitrile for each sample type. The mix was transferred to a new silica column, incubated for 1 min, and centrifuged. Columns with bound RNA were washed as described in [69], with an on-column digestion of DNA after the first washing (DNase I Set, Zymo Research), and eluted twice, first with 50 μL and then with 75 μL of RNase free-water for maximum yield.

For samples at T5, the previously described protocols were not effective and thus, a different protocol was used. 100 mg of liquid nitrogen grinded samples (three ECLs per treatment and two clones per ECL) were added to 2 mL tubes containing pre-heated (65 °C) extraction buffer (100 mM Tris, 30 mM EDTA, 2 M NaCl, 3% CTAB, 2% PVP-40, 2% PVPP, 4% β-mercaptoethanol). After vortexing, samples were incubated at 65 °C for 5 min and 800 μL of chloroform/isoamylalcohol (24:1, *v*/*v*) were added. Samples were vigorously mixed, incubated on ice for 2 min and centrifuged at 11,000 rpm for 10 min at 4 °C. The aqueous phase was saved and precipitated for 1 h at 4 °C by the addition of ¼ (*v*/*v*) LiCl (10 M). Then, samples were centrifuged at 11,000 for 30 min at 4 °C and the supernatant was discarded. Pellets were washed with 500 μL cold ethanol (70%), centrifuged for 5 min at 4 °C, air dried for 15 min and resuspended in 45 mL RNase free-water. After digestion of DNA using DNase I Set (Zymo Research), 600 μL of water and 700 μL of chloroform/isoamylalcohol (24:1, *v*/*v*) were added. The following steps (precipitation, washing…) were carried out as previously described and finally, RNA pellets were resuspended in 50 μL of RNAse free-water.

### 4.4. Expression Pattern of Stress-Related Genes

The expression level of 6 genes related to thermal [59] and drought stress [62] was determined by qRT-PCR from the RNA extracted at different stages of SE (T1: EMs after first subculture in semi-solid medium; T2: EMs after 4 weeks on proliferation medium; T3: mature Se’s; T4: completely germinated in vitro seedlings growing in glass jars; T5: apical needles of one-year-old somatic plants growing in the greenhouse). The genes analysed were the following:

- 50S RIBOSOMAL_L6_CHP (*50SR*): gene encoding a component of the ribosome, part of the translation machinery and responsible for the synthesis of proteins. Previously described as being temperature-responsive and important during early stress events and acclimation in radiata pine [59]. It has also been linked with the response to cytokinins [61], whose profiles were altered by high temperatures in previous studies [25,26].
- SUPEROXIDE DISMUTASE [Cu-Zn] (*SOD*): encoding an enzyme that catalyses the partitioning of the superoxide (O_2_^−^) radical. Heat provokes the synthesis of toxic reactive oxygen species (ROS) and plants require specific enzymes to detoxify these compounds. Previous studies have shown that oxidative stress is the main effect of high temperatures during early stages of radiata pine SE [24].
- TRANSCRIPTION FACTOR APFI (*APFI*): gene encoding a transcription factor that has been described as an excellent thermo-tolerance biomarker in radiata pine [59].
- HSP20 FAMILY PROTEIN (*HSP20*): gene coding for a protein member of the small heat shock protein family. These family of proteins have been widely documented as taking active part during heat response and memory acquisition in a great variety of plant species, including radiata pine [59].
- ADH_SF_ZN_TYPE (*ADH*): encodes an oxidoreductase that catalyses the oxidation of primary alcohols. Other studies have reported enhanced synthesis of this enzyme during heat stress in radiata pine and linked its accumulation with the production of secondary metabolites like flavonoids [59]. The presence of these last compounds was remarked in previous studies after heat exposure in EMs [24].
- DEHYDRATION INDUCED PROTEIN (*DI19*): gene coding for a member of the DI19 family of proteins. It has been proved their involvement during drought stress in *Arabidopsis* [62] and homologs have been reported in *P. radiata* [70].

cDNA was synthetized from 1000 ng of RNA using the PrimeScript RT Reagent Kit (Takara) and random hexamers as primers following the manufacturer’s instructions. Relative gene expression was measured by qPCR (StepOne Plus, Thermo Fisher Scientific). Three technical replicates were analysed per sample using ACTIN (*ACT*) as endogenous control. Primer efficiencies were estimated by template dilutions and the equation E = 10^(−1/slope)^, which gave values always greater than 1.81. Reactions were carried out in 20 µL containing 1 µg of cDNA, 0.3 µM of each primer and the SYBR green master mix (Takara Bio, Shiga, Japan). The PCR conditions were: initial denaturation at 95 °C for 20 s, followed by 40 cycles of 95 °C for 3 s and 60 °C for 30 s. The relative transcript levels were normalized using ACT, and the relative expression of each gene (R) was calculated on the basis of ΔC_t_ values [71] using the following formula: R = 2^−ΔCt^. Then, the fold change between expression values obtained at control treatment of 23 °C and treatments at 40 °C and 60 °C was calculated in logarithmic scale. Gene specific primers for all genes were as described by [59] and [70].

### 4.5. Statistical Analysis

To assess the effect of the treatments on both methylation/hydroxymethylation levels and gene expression values, a usual analysis of variance (ANOVA) was conducted. A Tukey’s post-hoc test (α = 0.05) was used for multiple comparisons. When required, the ECL was included in the model as a random effect to improve the fit and analyse the effect of treatments more accurately by accounting for heteroscedasticity in the data. When the analysis of variance did not fulfil the normality hypothesis a Kruskal–Wallis test was performed.

## Figures and Tables

**Table 1:** Methylation and hydroxymethylation rates of *Pinus radiata* proliferating embryonal masses collected at T2 and needles of one-year-old somatic plants (T5) originating from megagametophytes cultured under different temperature treatments (23 °C, 8 weeks; 40 °C, 4 h; 60 °C, 5 min). Data are presented as mean values ± SE. Significant differences among treatments at *p* < 0.05 are indicated by different letters.

T2		T5			
Treatment	5mC %	Treatment	5mC %	5hmC %	5hmC/5mC × 100
23 ° C, 8 weeks	39.28 ± 1.06 ^a^	23 ° C, 8 weeks	40.45 ± 0.53 ^a^	0.0175 ± 0.0024 ^a^	0.044 ± 0.0071 ^a^
40 ° C, 4 h	40.85 ± 0.36 ^a^	40 ° C, 4 h	40.39 ± 0.22 ^a^	0.0103 ± 0.0005 ^b^	0.026 ± 0.0012 ^b^
60 ° C, 5 min	37.82 ± 1.06 ^a^	60 ° C, 5 min	37.64 ± 0.33 ^b^	0.0101 ± 0.0003 ^b^	0.027 ± 0.0009 ^b^
